# More than words? Defining best practices in assessing surrogate understanding

**DOI:** 10.1093/atsscholar/aapag006

**Published:** 2026-01-30

**Authors:** Jason M Arnold, Melanie M Smith, Gordon J Wood, Julia H Vermylen, James M Walter

**Affiliations:** Department of Medicine, Northwestern University, Feinberg School of Medicine, Chicago, IL, United States; Department of Medicine, Northwestern University, Feinberg School of Medicine, Chicago, IL, United States; Department of Medicine, Northwestern University, Feinberg School of Medicine, Chicago, IL, United States; Department of Medicine, Northwestern University, Feinberg School of Medicine, Chicago, IL, United States; Department of Medicine, Northwestern University, Feinberg School of Medicine, Chicago, IL, United States

To the Editor:

We enjoyed reading the article “Differences in assessing surrogate understanding of the patient’s clinical situation” by Pecanac and Golden.^1^ The authors focus on the interplay between the phrasing of a clinician’s question and the surrogate’s response. The team recorded 27 clinician–surrogate conversations with “life or death implications” and analyzed them using “conversation analysis.” The clinician assessed surrogate understanding in only 10 of these recordings. Word choice in this subset of recordings seemed to matter, as asking what the surrogate “knew,” “heard,” or “understood” produced different responses. 

While definitive conclusions are limited by sample size, we agree that how a clinician phrases a question likely influences a surrogate’s response. There is value in understanding this interaction at the granular level explored by the authors. It is also important to acknowledge that assessing surrogate understanding should not be the work of a single question (let alone a single word). Rather, the essential skill is to determine the information important to the decision at hand and then to engage in a dialogue to establish a shared understanding with the patient or surrogate. Integral to a surrogate’s “understanding” is both an appreciation of the events that have occurred and a sense of what those events mean for the future. A surrogate may accurately state that their loved one with lung cancer has pneumonia and was placed on a ventilator. However, if a clinician does not assess what the surrogate understands about the likelihood of additional chemotherapy, ventilator liberation, or discharge home, true shared decision making will be difficult. This “future-oriented” view of surrogate understanding is largely absent from the excerpts provided in the article.

We have developed a simulation-based mastery learning (SBML) curriculum for goals-of-care discussions.^2^ In developing this curriculum, a multidisciplinary panel separated the assessment of surrogate understanding into 2 tasks: (1) elicit understanding of the current medical situation, and (2) elicit understanding of what the current medical situation means for the future. While the latter line of questioning is important, it is often skipped. In our work, only 50% of critical care medicine fellows and advanced practice providers demonstrated this skill during pretesting.^3^ Competency-based skills training can address this gap. During posttesting, 82% of learners assessed a surrogate’s understanding of the future.

In the study, there were several examples of surrogate expressions of fear and confusion that were not met with empathy by the clinician. Additionally, the authors point out that asking about understanding can be “tenuous, because it can be perceived as a test of knowledge.” Empathic responses could attenuate this perception and help uncover patient values. Consistently recognizing and empathically responding to emotion goes hand-in-hand with assessing understanding, especially of the implications for the future, since this is often “bad news.” In our work, only 18% of learners used an empathetic statement immediately after discussing such news during pretesting, whereas 86% did so after an SBML curriculum.^3^ Other studies have shown that there is often a discrepancy between clinician and family perceptions of whether emotional needs were addressed.^4^ Clinicians require feedback and practice to master this skill.^2^^,^^3^

The study by Pecanac and Golden provides important preliminary data to guide nuanced bedside communication. Words certainly matter. However, competence to lead these conversations likely hinges less on individual words and more on a clinician’s ability to consistently exhibit a core set of skills in every clinical encounter, including engaging in a dialogue that assesses understanding of the current situation and what it means for the future while also attending to the emotions these questions raise. We ­believe that these skills can be defined, taught, and assessed. 

## Supplementary Material

aapag006_Supplementary_Data

## References

[1] Pecanac KE , Golden BP. Differences in assessing surrogate understanding of the patient’s clinical situation. ATS Sch. 2025;6:488-501. 10.34197/ats-scholar.2024-0149OC 40833441 PMC12712825

[2] Walter JM , Smith MM, Einstein N, Cohen ER, Wood GJ, Vermylen JH. Development of a simulation-based mastery learning curriculum for late goals of care discussions. J Pain Symptom Manage. 2024;68:e54-e61. 10.1016/j.jpainsymman.2024.03.020 38527655

[3] Smith MM , Walter JM, Munger NK, Cohen ER, Vermylen JH, Wood GJ. Excellence for all: simulation-based mastery learning for ICU goals of care conversations. Chest. 2025;168:1186-1189. 10.1016/j.chest.2025.06.004 40553815

[4] Mahendiran M , Yeung H, Rossi S, Khosravani H, Perri GA. Evaluating the effectiveness of the SPIKES model to break bad news—a systematic review. Am J Hosp Palliat Care. 2023;40:1231-1260. 10.1177/10499091221146296 36779374

